# Genome-guided purification and characterization of polymyxin A1 from *Paenibacillus thiaminolyticus* SY20: A rarely explored member of polymyxins

**DOI:** 10.3389/fmicb.2022.962507

**Published:** 2022-11-14

**Authors:** Ya-ping Wu, Dong-mei Liu, Ming-hua Liang, Yan-yan Huang, Jin Lin, Lan-fang Xiao

**Affiliations:** ^1^School of Food Science and Engineering, South China University of Technology, Guangzhou, China; ^2^College of Food Engineering, Zhangzhou Institute of Technology, Zhangzhou, China

**Keywords:** polymyxin A1, antimicrobial agent, genome mining, Gram-negative, *Paenibacillus thiaminolyticus*

## Abstract

Polymyxin A1 was a rarely investigated member in the polymyxins family produced by *Bacillus aerosporus*. As a cyclic non-ribosomal lipopeptide, it was purified from *Paenibacillus thiaminolyticus* for the first time. The producing strain SY20 was screened from Chinese natural fermented bamboo shoots and identified as *P. thiaminolyticus* SY20 using 16S rRNA homology along with whole genome sequencing. The optimum incubation time was 32 h by the growth kinetics of antimicrobial agent production. The proteinaceous nature of antimicrobial agents was characterized according to the physicochemical properties of the cell-free supernatant. Subsequently, the active antimicrobial agent was purified from the supernatant using ammonium sulfate–graded precipitation, ion-exchange chromatography, and C_18_-H chromatography. The active agent was identified as polymyxin A1 with a molecular weight 1156.7 Da and antimicrobial activity mainly against Gram-negative bacteria. The molecular structure, a cyclic heptapeptide and a tripeptide side chain acylated by a fatty acid at the amino terminus, was elucidated using the combination of liquid chromatography-tandem mass spectrometry (LC-MS/MS), matrix-assisted laser desorption ionization–time of flight mass spectrometry (MALDI-TOF MS), amino acid analysis, and whole genome mining tool. Meanwhile, the biosynthetic gene cluster of polymyxin A1 including five open reading frames (ORFs) was demonstrated in the genome. The compound should be further explored for its efficacy and toxicity *in vivo* to develop its application.

## Introduction

An enormous and growing threat aroused by multidrug-resistant (MDR) pathogenic bacterial infections posed a severe challenge to human health. With the ineffective use of β-lactam, quinolone, or aminoglycoside in killing MDR pathogenic bacteria and slower growth in discovering new drugs, the polymyxins are gradually considered as the last line of defense for MDR pathogenic bacteria against almost all other currently available antibiotics (Han et al., 2019; Yin et al., 2020). Polymyxins are a family of cyclic non-ribosomal lipopeptides discovered more than 70 years ago and produced by the widely distributed soil bacterium *Paenibacillus polymyxa* (Bergen et al., 2015; Velkov et al., 2019). They are usually used for the treatment of Gram-negative bacterial infections such as *Pseudomonas aeruginosa, Acinetobacter baumannii*, and *Klebsiella pneumoniae* (Bergen et al., 2015; Velkov et al., 2019; Pajor et al., 2020a). At least 25 chemically distinct members have been found to make up the polymyxins family, among which polymyxin B, polymyxin E (colistin), and their derivatives are commercially used as clinical medicines against the infection of MDR pathogenic bacteria (Velkov et al., 2019). However, the inevitable nephrotoxicity and neurotoxicity served as a primary limitation to affect their parenteral administration (Yu et al., 2015). Polymyxin A, initially called “Aerosporin,” was produced by *Bacillus aerosporus* isolated from the soil of a market garden in Surry (Ainsworth et al., 1947). It conforms the basic pattern of polymyxins that possesses a cyclic heptapeptide and a tripeptide side chain acylated by a fatty acid at the amino terminus (Wilkinson and Lowe, 1966; Velkov et al., 2019). Two variants (A1 and A2) were distinguished with the difference between fatty acyl groups, which were identified as 6-methyloctanoic acid (C_9_H_17_O) and 6-methyl-heptanoic acid (C_8_H_15_O), respectively (Tambadou et al., 2015). Six non-proteinaceous amino acids, 2,4-diaminobutyric acid (Dab), constitute the amino acids in the formula, among which the Dab at position 4 forms an amide linkage with the C-terminal threonine.

The genus *Paenibacillus* was distinguished from *Bacillus* by an extensive comparative analysis of 16S rRNA sequences in 1993 (Cotta et al., 2012). Bacteria belonging to the genus are widely distributed in the environment and produce a variety of active substances (Guo et al., 2012; Baindara et al., 2016), which involved in antibacterial including lantibiotics, polyketides, lipopeptides, macrolide, and peptide–polyketide (He et al., 2007; Wu et al., 2011; Guo et al., 2012).

This study aimed to screen a novel microbial strain with the potency of producing antimicrobial agents against foodborne pathogens or human pathogens. Since the purification and identification of an antimicrobial agent require laborious procedures if followed the classical methods (Lebedeva et al., 2020), genome mining approaches that explore both DNA and peptide databases have provided a rationale for targeted isolation of active agent from complex protein mixtures (Baindara et al., 2020). In addition, mass spectrometry also facilitates the identification and quantification of novel antimicrobial agents. In our work, we reported a strain isolated from the sour bamboo shoots sample with antagonistic activity mainly against Gram-negative bacteria. Its taxonomic affiliation was confirmed by 16S rRNA and whole genome sequencing, then it was named *Paenibacillus thiaminolyticus* SY20. The growth conditions of the strain were optimized to achieve maximum antimicrobial production, and the physicochemical properties of the cell-free supernatant were evaluated to characterize the antimicrobial agents produced by the strain. Subsequently, the active agent from the supernatant was purified by ammonium sulfate fractional precipitation, ion-exchange chromatography, and reversed-phase high-performance liquid chromatography (RT-HPLC). Finally, the active agent was identified as polymyxin A1 by liquid chromatography–tandem mass spectrometry (LC-MS/MS), matrix-assisted laser desorption ionization–time of flight mass spectrometry (MALDI-TOF MS), amino acid analysis together with whole genome mining for biosynthetic gene clusters. To the best of our knowledge, this was the first time that polymyxin A1 has been isolated from *P. thiaminolyticus*, providing a systematic and effective approach to purifying and identifying the antimicrobial agent.

## Materials and methods

### Bacterial strains and culture media

*Staphylococcus aureus* (RN 4220) and *Escherichia coli* (ATCC 25922) were the primary indicator bacteria. *Samomella enteritidis* (CCTCC AB 94018), *Klebsiella Pneumoniae* (ATCC 10031), *Enterobacter Sakazakii* (ATCC 29544), *Vibrio parahaemolyticus* (ATCC 10031), *Listeria monocytogenes* (ATCC 19115), *Pseudomonas aeruginosa* PAO1, *Salmonella typhimurium* (ATCC 14028), *Shewanella putrefaciens* (ATCC 8071), and *Pseudomonas fluorescens* (ATCC 13525) were purchased from China Center of Industrial Culture Collection (CICC). *Lactiplantibacillus plantarum* DMDL 9010, *Lacticaseibacillus rhamnosus* B1107, *Bacillus coagulans* 13002, *Bacillus amyloliquefaciens* K1, and *Bacillus licheniformis* SG18 were isolated and preserved in Guangdong Microbial Culture Collection center (GDMCC). All strains were used as indicator bacteria for antimicrobial spectrum detection.

The medium of tryptone-glucose-yeast extract (TGYE) and nutritional broth (NB) was used for screening the antimicrobial strains from the sample; tryptic soy broth (TSB), brain heart infusion (BHI), Luria-Bertani (LB), and de Man, Rogosa and Sharpe (MRS) purchased from Sangon Biotech (Shanghai) were used as the cultures for different indicator bacteria.

### Antimicrobial activity determination

The agar well diffusion method was applied to determine the antimicrobial activity described by Wu et al. (2018). Briefly, each indicator bacteria was cultivated to the logarithmic growth phase. An appropriate amount of indicator bacteria liquid was injected into the corresponding medium containing 0.75% agar with the final bacterial inoculum of 5 × 10^5^ CFU/ml. The mixture was poured into sterile plates, and several wells were made after solidification. Fifty microliters of tested samples were added into each well, and the antimicrobial activity was expressed as the diameter of inhibition zone after incubating at 37°C for 8 h.

### Screening and identification of the antimicrobial strains

The sour bamboo shoots sample purchased from the Xiangya center market in Zhangzhou, Fujian Province (China), was used to screen the microorganisms that produce antimicrobial agents (He et al., 2007). Briefly, samples were cut into small pieces and suspended in sterilized saline. After shaking to homogenize, the suspensions were serially diluted. Aliquots (150 μl) were spread on TGYE and NB agar and then were incubated at 28°C. The strains were singled out and inoculated into LB medium for 48 h to evaluate their antimicrobial potential. An isolated strain named SY20, whose fermentation supernatant showed antimicrobial activity, was selected for further biological identification. The 16S rRNA sequence of SY20 was aligned in NCBI Genbank. The whole genome was sequenced by Beijing Genomics Institute (BGI, China).

### Growth kinetics of antimicrobial agent production

The activated SY20 strain was inoculated into a 1 L triangular flask containing 500 ml LB and incubated at 37°C with continuous shaking at 180 r/min. The culture samples were taken every 4 h to measure their pH, OD_600_, and the antimicrobial activity of cell-free supernatant against *E. coli* ATCC 25922 (Pajor et al., 2020b).

### Effect of temperature, pH, enzymes, detergents, and metal ions on the antimicrobial activity of the cell-free supernatant

To characterize the properties of the active agents in the cell-free supernatant and further guide the purification process, the antagonistic activity of the supernatant obtained in the optimum incubation time was assessed after exposure to different pH values, temperatures, enzymes, detergents, and metal ions (Wu et al., 2018). The cell-free supernatant was adjusted to a pH range from 3.0 to 11.0 with hydrochloric acid (HCl) and sodium hydroxide (NaOH), respectively. The antimicrobial activity was assayed with the medium in the same pH value as the blank controls after 6 h of incubation at room temperature. To investigate the effect of thermostability on the antimicrobial activity of the supernatant, aliquots were exposed at 4, 25, 40, 60, 80, 100, and 121°C for 30 min, and the residual antimicrobial activity was tested. The sensitivity of the supernatant to enzymatic degradation was evaluated using catalase, trypsin, α-amylase, proteinase K, pepsin, lipase, and papain (all purchased from Sigma-Aldrich, USA) with an ultimate concentration of 1 mg/ml under the appropriate conditions. Reaction mixtures were incubated for 3 h, and then antagonistic activity was assayed with enzyme solutions and the supernatant without enzymes as controls, respectively. To understand the sensitivity to detergents, the supernatant was exposed to 0.01% SDS, 1% Triton-100, Tween-20, Tween-80, urea, and EDTA at room temperature for 4 h, respectively, then the residual antimicrobial activity was checked as the supernatant without treatment of detergents was used as a positive control. In a similar way, the supernatant was mixed with K^+^, Na^+^, Mg^2+^, Ca^2+^, Mn^2+^, Al^3+^, and Fe^3+^ solutions at a final concentration of 0.1 M, 0.1 M, 0.1 M, 0.1 M, 0.01 M, 0.0025 M, and 0.05 M, respectively, to examine the effect of metal ions. The mixtures were incubated at room temperature for 6 h before testing for antimicrobial activity. The supernatant without ion solution treatment was used as the positive control and ion solutions as blank controls.

### Purification of the antimicrobial agent from the cell-free supernatant

The crude extract (CE) with antimicrobial activity was obtained by ammonium sulfate–graded precipitation from the culture supernatant of SY20 (Wu et al., 2018; Pajor et al., 2020b). Briefly, the 1 L supernatant was subjected to precipitation. Firstly, the solid ammonium sulfate was added to the supernatant to 50% saturation and left for 6 h at 4°C, then the suspension was centrifuged (10,000 g, 20 min) to separate the precipitate and solution. The precipitate (50%) was collected and dissolved with 20 ml of distilled water. The solution was further treated with 65% (NH_4_)_2_SO_4_ saturation. The precipitate (65%) was obtained as mentioned above. Finally, the solution was additionally added to 100% (NH_4_)_2_SO_4_ saturation, and the precipitate (100%) was obtained in the same way. The antimicrobial activity of the re-dissolved precipitations was checked, and the active one was dialyzed against distilled water at 4°C until the ammonium sulfate was removed entirely. The CE with antimicrobial activity was harvested by lyophilization.

The purification of the CE was performed using cationic exchange and reversed-phase chromatography. The CE was re-dissolved in 20 mM Tris-HCl (pH 7.2) buffer and sterilized with a 0.22 μm membrane. A 10-ml CE sample was first loaded onto the CM Sepharose Fast Flow column (16 mm × 100 mm, GE), which was equipped with an AKTA Pure system (GE). The column was preconditioned with buffer A (20 mM Tris-HCl, pH 7.2), then the gradient elutions with 30, 60, and 100% buffer B (20 mM Tris-HCl and 1 M NaCl) were carried out and monitored at 214 and 280 nm UV absorption. The antimicrobial activity of the eluted fractions was determined with *E. coli* ATCC 25922 as an indicator. The fraction, which was checked to possess antimicrobial activity, was dialysis to desalt with a 500 Da cutoff membrane and concentrated by lyophilization.

The active fraction dissolved in water was further separated on an RP-HPLC system (Agilent 1260). A 100-μl volume of active solution was injected into C_18_-H (4.6 × 250 mm, 5 μm, Amethyst), then eluted using mobile phase solution which contained 22% acetonitrile and 78% Na_2_SO_4_ solution (31.4 mM, adjusted the pH value to 2.5 with H_3_PO_4_) at the flow rate of 1 ml/min. All the peaks monitored at 214 nm were collected and tested for the antagonistic activity with *E. coli* ATCC 25922.

### Identification of the antimicrobial agent by mass spectrometry

The active peak purified by C_18_-H column was dissolved with water after dialysis and lyophilization. Then the sample was subjected to MALDI-TOF MS analysis on a Bruker autoflex III smartbean mass spectrometer (Bruker Daltonics Inc., Billerica, MA) and operated in reflection positive ion mode at an accelerating voltage of 28 kV.

The sample was analyzed by LC-MS/MS for molecular weight and amino acid sequence determination. Samples were separated on a capillary column (2.1 × 50 mm, SB-C_18_ RRHD, Agilent) using an Agilent 1290 HPLC system, and a capillary voltage of 3.5 kV was used. Tandem mass spectrometry was performed on a maXis impact mass spectrometer (Bruker Daltonics Inc., Billerica, MA) equipped with an ESI source, operated in positive-ion mode. The MS/MS spectrum was acquired with an ESI source operated with a spray voltage of 2 kV, and the scan sequence of the mass spectrometer was performed from 50 to 2,000 m/z.

### Elucidation of the antimicrobial agent by whole genome mining tool

AntiSMASH 5^1^ was used to detect non-ribosomal peptide synthetases (NRPS) and the potential bacteriocin biosynthetic gene clusters (Weber et al., 2015). The NRPS/PKS (polyketide synthase) monomer structure and prediction of possible NRPS substrate were carried out by NRPSpredictor2^2^ and NPRSsp.^3^

### Verification of the antimicrobial agent by amino acid composition analysis and antimicrobial assay

For amino acid composition analysis, the amino analysis system L-8900 (Hitachi) was employed as per the manual of operator and the reference of method (Jangra et al., 2018). In brief, a sufficient amount of active agent was hydrolyzed by 6 N HCl in a sealed ampoule at 110°C for 24 h. Afterward, the solvent was evaporated using a vacuum drying chamber (Yiheng, Shanghai) and resuspended in disodium hydrogen phosphate buffer (pH3.3). Analysis was done by the amino analysis system L-8900 (Hitachi) according to the methods provided by the manufacturer.

Lipopolysaccharide (LPS) is a component of the outer wall of Gram-negative bacterial cells (Huang and Yousef, 2014; Abdelhamid and Yousef, 2019). LPS derived from *E. coli* 0111: B4 was purchased from Sigma. A certain amount of LPS were added to the active agent and the supernatant, respectively, then incubated for 1 h at room temperature to determine the antimicrobial activity against *E. coli* ATCC 25922. The change in antimicrobial activity was observed with or without LPS in the samples. In addition, the antimicrobial spectrum of the supernatant and the active agent was determined using the agar well diffusion method described in section “Antimicrobial activity determination.”

### Statistical analysis

The statistical analysis of the data was conducted by SPSS 22.0. Three parallels were repeated for each experiment, and experimental differences were determined using a one-way ANOVA and Duncan test at 0.05 levels.

## Results

### Isolation and identification of an antimicrobial strain

About three hundred and fifty strains of bacteria were isolated from the sour bamboo shoots sample. The supernatants of all strains were tested the antimicrobial activity with *S. aureus* RN 4220, *E. coli* ATCC 25922, and *S. enteritidis* CCTCC AB 94018. A strain designated SY20 showed antagonistic activity against the three indicators. 16S rRNA genomic analysis of SY20 shared high similarity with *Paenibacillus thiaminolyticus* (99%), and the whole genome sequencing confirmed the result, which was submitted to NCBI with the accession number CP106992. Thus, it was given the name *Paenibacillus thiaminolyticus* SY20.

### Growth kinetics of antimicrobial agent production

**FIGURE 1:**
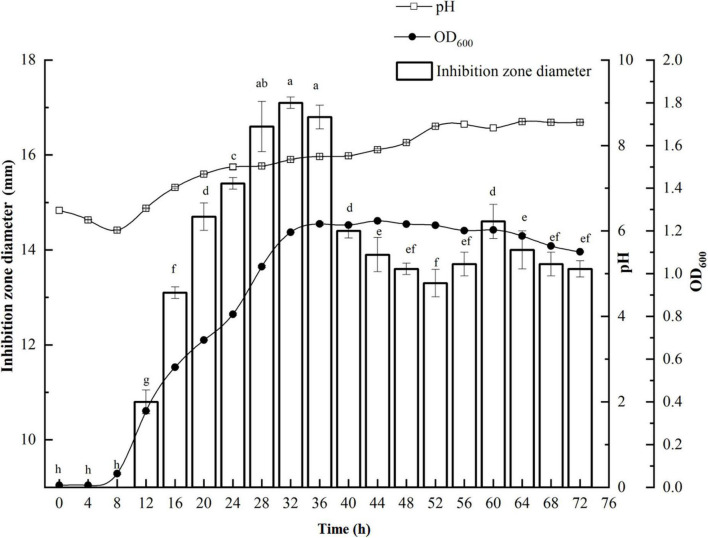
Growth kinetics of *P. thiaminolyticus* SY20 against *E. coli* ATCC 25922. The line chart represents the change in the bacterial density and pH in the fermentation broth with the culture time, respectively. The histogram represents the antimicrobial activity of the fermentation supernatant against *E. coli* ATCC 25922, inhibition zone diameter is expressed as mean ± standard deviation (*n* = 3), each column followed by the different letter is significantly different at the 5% level.

*P. thiaminolyticus* SY20 initiated the synthesis of antimicrobial agents against *E. coli* ATCC 25922 from 12 h at the early logarithmic phase (Figure 1). Maximum antimicrobial activity against *E. coli* ATCC 25922 (17.1 mm) was found after 32 h incubation during the end of the logarithmic phase. Then the antimicrobial activity was gradually decreased with the increase in incubation time but did not disappear until 72 h. At the same time, the value of pH changed from weak acidity to weak alkaline. The optimum incubation time for the productivity of antimicrobial agents against *E. coli* ATCC 25922 was selected at 32 h.

### Characterization of the physicochemical properties of the supernatant

**FIGURE 2:**
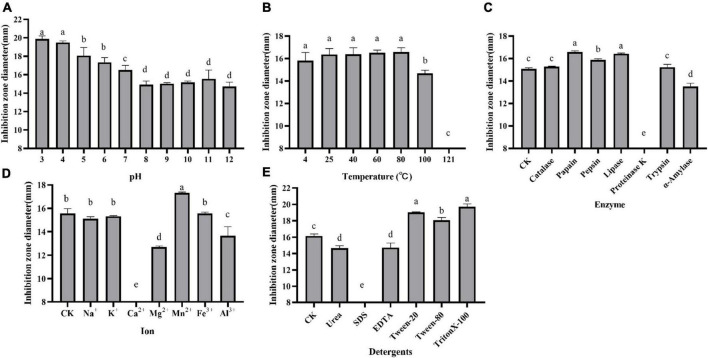
Effect of pH **(A)**, temperature **(B)**, enzymes **(C)**, metal ions **(D)**, and detergents **(E)** on the antagonistic activity of supernatant against *E. coli* ATCC 25922. Inhibition zone diameter is expressed as the mean of three replicates, and the vertical bar indicates the standard deviation, each column followed by the same letter is not significantly different at the 5% level.

The effect of pH, temperature, enzymes, detergents, and metal ions on the antagonistic activity of the supernatant is shown in Figure 2. The supernatant was active in the acidic conditions and gradually increased when the pH value decreased. However, the antimicrobial activity was slashed in alkaline conditions but retained at pH 12.0 (Figure 2A). The antagonistic activity remained when the supernatant was exposed to 80°C for 30 min. But the activity was lost after autoclaving for 30 min at 121°C (Figure 2B). It was basically not impacted by the treatment of catalase, trypsin, pepsin, lipase, and papain but partially affected by α-amylase and totally lost activity by proteinase K (Figure 2C). Different mental irons on antimicrobial activity demonstrated that it was stable at 0.1 M Na^+^, K^+^, and 0.05 M Fe^3+^ and slightly improved by 0.01 M Mn^2+^. However, the activity was obviously inhibited by 0.1 M Mg^2+^ and 0.0025 M Al^3+^ even inactivated by 0.1 M Ca^2+^ (Figure 2D). The antimicrobial activity of the supernatant was slightly inhibited by 1% urea and EDTA but was found to increase with the addition of 1% Triton-100, Tween-20, and Tween-80. Notably, the antagonistic activity was lost after treatment with 0.01% SDS (Figure 2E).

### Preparation and purification of antimicrobial agent

**FIGURE 3:**
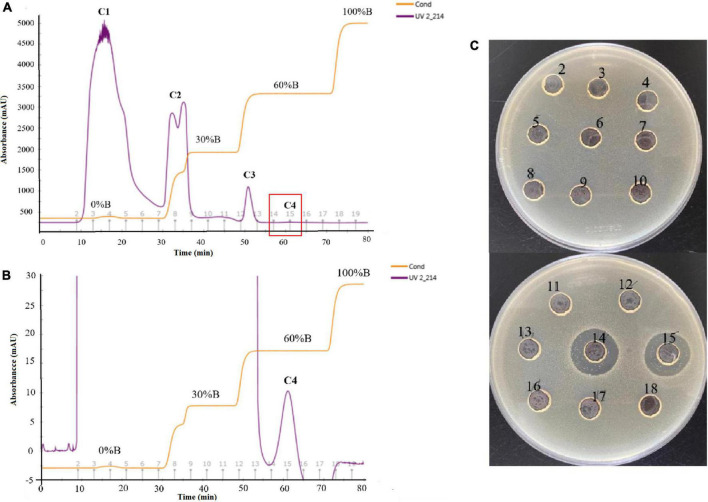
Purification of the antimicrobial agent from the crude extract by CM Sepharose Fast Flow column **(A)**, zoom in locally on the peak C4 **(B)**, and determination of the antimicrobial activity of different tubes against *E. coli* ATCC 25922 **(C)**. The elution components were collected with 8 ml per tube (the serial number of each tube was marked on the abscissa from 2 to 19), three distinct peaks C1 (tubes 2–6), C2 (tubes 7–9), C3 (tube12), and one not obvious peaks C4 (tubes 14 and 15) were isolated, zooming out the range of the longitudinal axis could exhibit the C4 clearly **(B)**.

**FIGURE 4:**
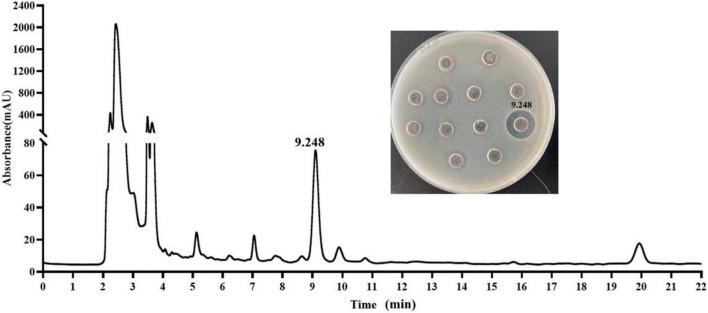
Purification of the antimicrobial agent from the fraction C4 by C_18_-H column and determination of the antimicrobial activity of the peaks against *E. coli* ATCC 25922.

The crude extract (CE) was prepared by ammonium sulfate–graded precipitation. The precipitations (precipitated by 65∼100% saturation) exhibited the best antagonistic activity against *E. coli* ATCC 25922 (Supplementary Figure 1). After being dialyzed and lyophilized, the CE was prepared and then further purified by ion-exchange chromatography and RP-HPLC. The CE, re-dissolved in 20 mM Tris-HCl buffer (pH 7.2), was first loaded onto the CM Sepharose Fast Flow column. The elution components were collected with 8 ml per tube. Three peaks C1 (tubes 2–6), C2 (tubes 7–9), and C3 (tube12), as well as one not obvious C4 (tubes 14 and 15), were isolated (Figure 3A). The C4 eluted by 60% buffer B showed a distinct UV absorption peak at 214 nm when it was partially amplified (Figure 3B) and exhibited strong antagonistic activity against *E. coli* ATCC 25922 (Figure 3C). The active fraction C4 was dialyzed and further separated on an RP-HPLC system with the C_18_-H column. An active peak at the retention time of 9.248 min was observed, and the collected fraction exhibited antagonistic activity by agar well diffusion method, as shown in Figure 4, which suggested the active agent was purified.

### Characterization of the antimicrobial agent by LC-MS and matrix-assisted laser desorption ionization–time of flight mass spectrometry

**FIGURE 5:**
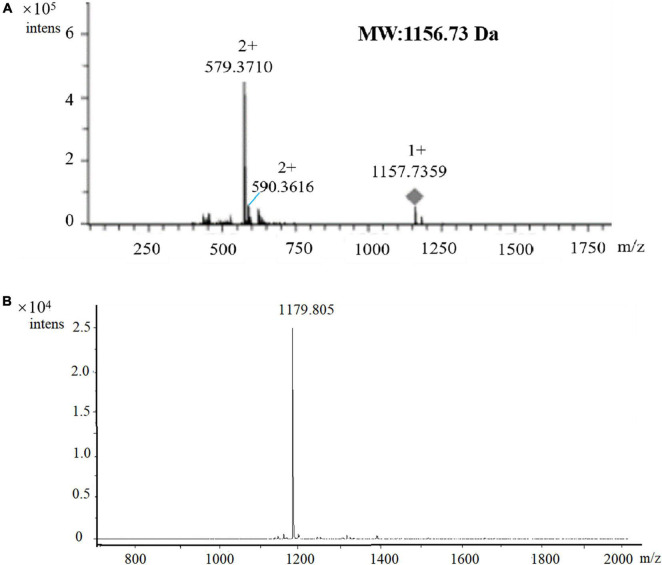
Verification of the molecular mass and purity of the antimicrobial agent by LC-MS **(A)** and MALDI-TOF MS **(B)** analysis. m/z = 1179.805, which was inferred with [M + Na]^+^, the molecular mass of 1156.83 Da was calculated.

The HPLC-purified agent was subjected to LC-MS using a maXis impact mass spectrometer. The primary mass spectrometry analysis found that the compound with the retention time of 9.284 min had a high-resolution multi-charge ion [M + 2H]^2+^ value of 579.3710, the other multi-charge ion [M + H + Na]^2+^ value of 590.3616, and a molecular ion [M + H]^+^ of 1157.7359, which could be determined that the molecular mass of the active agent was 1156.73 Da (Figure 5A). Subsequently, MALDI-TOF MS was performed for molecular mass and purity. Only one peak with m/z at 1179.805 was observed (Figure 5B), which was inferred with [M + Na]^+^, and the molecular mass of 1156.83 Da was calculated, which was basically consistent with that of LC-MS, and also revealed the active agent as a pure compound.

### Elucidation of the antimicrobial agent combined with whole genome mining

**FIGURE 6:**
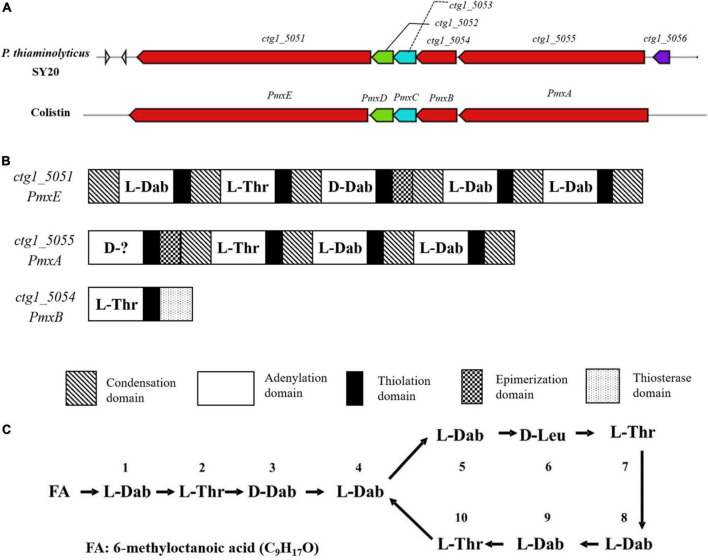
Analysis of NRPS gene cluster and the structure of polymyxin A1 from *P. thiaminolyticus* SY20. **(A)** The *pmx* gene cluster of colistin and a hybrid NRPS gene cluster in *P. thiaminolyticus* SY20; **(B)** genetic structure of the hybrid NRPS gene cluster and domain organization of every modular; **(C)** Primary structure of polymyxin A1, FA: 6-methyloctanoic acid.

To further identify the active agent, the whole genome of *P. thiaminolyticus* SY20 was loaded onto the AntiSMASH 5 to detect NRPS and gene clusters of ribosomally synthesized bacteriocins. A hybrid NRPS gene cluster, spanning a 40.69 kb region, was found in the whole genome, which shared high similarity with the gene cluster of colistin (polymyxin E1) from *Paenibacillus alvei* B-LR (Tambadou et al., 2015), as shown in Figure 6A. Colistin is a lipopeptide antibiotic produced by strains of *Paenibacillus polymyxa* and widely used to treat infections caused by multi-resistant harmful bacteria (Matzneller et al., 2015; Tambadou et al., 2015). The *pmx* gene clusters possessed five open reading frames (ORFs) named *PmxA, PmxB, PmxC, PmxD*, and *PmxE*, with three genes (*Pmx*A, B, and E) encoding NRPS and two genes (*Pmx*C and D) encoding putative ABC transporters, which were mainly involved in the externalization or resistance of the antibiotics (Tambadou et al., 2015). Five ORFs with the same transcription orientation were included in the hybrid NRPS gene cluster corresponding with those of the *pmx*, among which the ORFs *ctgl-5051, ctgl-5052, ctgl-5053, ctgl-5054*, and *ctgl-5055* shared 96, 98, 95, 95, and 91% identities with *PmxE, PmxD, PmxC, PmxB*, and *PmxA*, respectively (Figure 6A). Hence, we deduced the hybrid NRPS gene cluster might encode polymyxin synthetase, the ORF *ctgl-5053* and *ctgl-5054* encode membrane transporters, while *ctgl-5051*, ctg*l-5052*, and ctg*l-5055* encode polymyxin synthetase.

NRPS is produced by a multienzyme complex with modular structures (Sukhanova et al., 2018). Each module is responsible for assembling one or more specific amino acids into the final product. The typical module of an NRPS consists of different domains, such as adenylation (A), thiolation (T), condensation (C), epimerization (E), and thioesterase (TE) domains (Kim et al., 2015). The A domain plays the role of selecting and activating the amino acid substrate; once the correct substrate is selected, the T domain is responsible for transporting the substrate to the catalytic center through covalent bond binding, and then the C domain catalyzes the formation of peptide bonds. In addition to these core domains, the thioesterase domain (TE) plays the role of terminating enzyme reactions and releasing products, and the E domain is mainly responsible for the conversion of L-amino acids to D-amino acids (Choi et al., 2009; Galea et al., 2017). The hybrid NRPS encoding cluster consisted of ten modules that were subjected to NPRSsp (see text footnote 3) for monomer predictions, among which the *ctgl-5051* contained five modules encoding the synthesis of Dab-Thr-(D)Dab-Dab-Dab, the *ctgl-5055* contained four modules encoding the synthesis of (D)X-Thr-Dab-Dab, and the *ctgl-5054* contained a module encoding the synthesis of Thr (Figure 6B).

Although the first amino acid of the *ctgl-5055* gene failed to predict, it was certain that the active agent may belong to the polymyxin family according to the amino acid sequence encoded by these modules. Combined with the molecular weight 1156.73 Da measured by LC-MS and MALDI-TOF MS, the polymyxin A1 was suggested for the purified active agent, which was reported by Wilkinson in 1966 (Wilkinson and Lowe, 1966), and the first amino acid candidate of the *ctgl-5055* gene was D-Leu. The structure of polymyxin A1 is a cyclic heptapeptide with a tripeptide side chain acylated by a fatty acid at amino terminus as shown in Figure 6C. It possessed a theoretical mass of 1,157 Da, which was exactly in accord with that of our results detected by LC-MS or MALDI-TOF MS. Simultaneously, the amino acid sequence encoding by *ctgl-5051, ctgl-5055*, and *ctgl-5054* was completely contained in the polymyxin A1 molecular formula with *ctgl-5051* coding position 1–5, *ctgl-5055* coding position 6–9, and *ctgl-5054* coding position 10, respectively.

### Verification of the antimicrobial agent by liquid chromatography-tandem mass spectrometry and amino acid analysis

**FIGURE 7:**
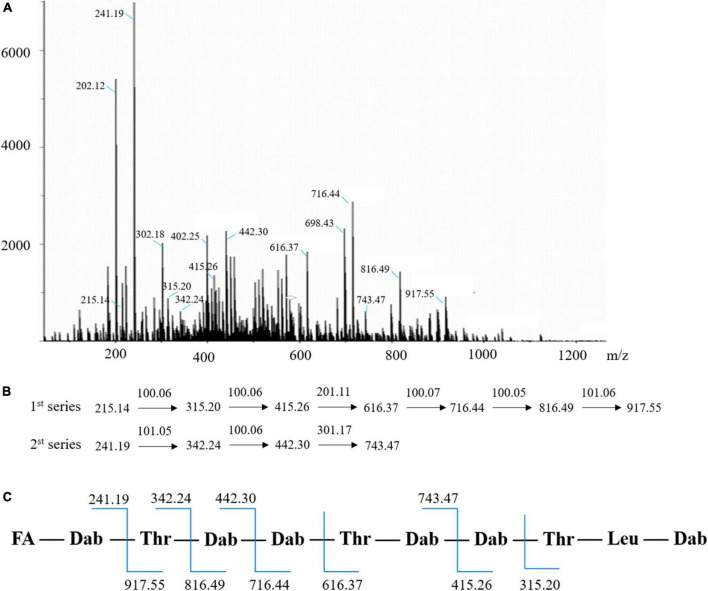
Verification of the antimicrobial agent as polymyxin A1 by LC-MS/MS and amino acid analysis. **(A)** MS/MS spectra acquired for the m/z = 1157.7359; **(B)** fragment ions (m/z); **(C)** proposed linear structure for the antimicrobial agent polymyxin A1. FA: 6-methyloctanoic acid (C_9_H_17_O).

MS/MS analyses were performed in order to confirm the inference of antimicrobial agent as the polymyxin A1 (Figure 7A). A number of amino acid residues with an experimental mass of 100.06 Da suggested the presence of 2,4-diaminobutyric acid (Dab) moieties in the structure (Figure 7B; Martin et al., 2003). The cationic Dab residues with a molecular formula of C_4_H_8_N_2_O were ubiquitous within the cyclic heptapeptide and side chain groups on polymyxin, which played a key role in polymyxin’s antimicrobial activity and the highly cationic nature of molecule (Yu et al., 2015). It was known that the polymyxin contained a distinct fatty acyl group (FA) that varies in length from 7 to 9 carbons and has been identified including (S)-6-methyloctanoyl, 6-methylheptanoyl, octanoyl, heptanoyl, nonanoyl, and 3-hydroxy-6-methyloctanoyl (Velkov et al., 2019). A distinct peak 241.19 in MS/MS was deduced to be FA-Dab, hence, the fatty acyl group with a mass of 141.13 Da was characterized as 6-methyloctanoyl (C_9_H_17_O) which coincided with the one in polymyxin A1 (Figures 7A,C). Simultaneously, a lot of fragment ions were identified in accordance with the composition of the polymyxin A1 (Figure 7C). The amino acid analysis also supported the MS/MS data, and the amino acid composition matched with polymyxin A1 (Supplementary Figure 2). Furthermore, the antimicrobial determination further supported the result. The antimicrobial activity against *E. coli* ATCC 25922 disappeared when a certain amount of LPS were added to the active agent and the supernatant, respectively (Supplementary Figure 3), which was consistent with the antimicrobial mechanism of polymyxin and suggested the antimicrobial agent belonged to the family.

### Antimicrobial spectrum of the supernatant and the active agent

The antimicrobial spectrum of the supernatant and the active agent was determined using the agar well diffusion method, and the results are shown in Supplementary Table 1. Antimicrobial assays showed that they could inhibit numerous Gram-negative species, including *E. coli* ATCC 25922, *S. enteritidis* CCTCC AB 94018, *K. pneumoniae* ATCC 10031, *E. sakazakii* ATCC 29544, *V. parahaemolyticus* ATCC 10031, *Psychrobacter pulmonis, Pseudomonas aeruginosa* PAO1, and *S. typhimurium* ATCC 14028 but failed to inhibit *S. putrefaciens* ATCC 8071 and *P. fluorescens* ATCC 13525. As for Gram-positive bacteria, the active agent isolated was ineffective against all tested bacteria, but the supernatant was effective against *B. subtilis, L. plantarum* DMDL 9010, *S. aureus* RN4220, *L. rhamnosus* B1107, and *B. licheniformis* SG18. In addition, the supernatant and active agent failed to inhibit *P. thiaminolyticus* SY20 itself.

## Discussion

*Paenibacillus* genus is known for its potential to produce a wide range of structurally diverse antimicrobial compounds, including bacteriocins, non-ribosomal lipopeptides, polyketides, peptide–polyketide hybrids, and even a wide range of volatile organic compounds (Grady et al., 2016; Lebedeva et al., 2020). Therefore, the supernatant of *P. thiaminolyticus* SY20 was used to determine the physical and chemical properties, which were regarded as an essential task for characterizing the antimicrobial agents and contributed to the subsequent purification. For example, the supernatant was sensitive to proteinase K (Figure 2C), which indicated the proteinaceous nature of antimicrobial agents produced by *P. thiaminolyticus* SY20 (Perumal and Venkatesan, 2017). The antimicrobial activity of the supernatant was damaged by SDS (Figure 2E), which may be attributed to the destruction of the spatial structure of proteinaceous antimicrobial agents. The supernatant was found to be active in acidic conditions and gradually slashed in alkaline ones (Figure 2A), which could be used as a reference for buffer selection in the purification process. The thermostability of the supernatant suggested that purification could be carried out in a wide temperature range.

Outstandingly, the antimicrobial activity was obviously inhibited by Mg^2+^ and even inactivated by Ca^2+^ (Figure 2D), which inspired that contact with these ions should be avoided during purification. In addition, ionic sensitivity also implied that the active agent belonged to the polymyxin family. As previously reported, the increasing concentrations of Ca^2+^ and Mg^2+^ were already shown to hamper or abolish the antibacterial efficacy of polymyxins (Davis et al., 1971; Matzneller et al., 2015). One of the antimicrobial mechanisms of polymyxin against Gram-negative bacteria was believed that it interacted electrostatically with the outer membrane of Gram-negative bacteria and killed bacteria through membrane lysis (Yu et al., 2015). Some divalent cations, such as Ca^2+^ and Mg^2+^, usually serve as a bridge between the adjacent lipopolysaccharide (LPS) molecules to stabilize the monolayer (Velkov et al., 2016). However, polymyxins could selectively bind to LPS through the positively charged Dab residues by displacing divalent cations (Ca^2+^ and Mg^2+^) and led to the destruction of the integrity of both the outer and the cytoplasmic membrane, ultimately leading to cell death (Yun et al., 2017). It was speculated that the addition of Ca^2+^ and Mg^2+^ irons might compete with polymyxin A1 in the binding of the LPS, thus impairing the antimicrobial effect of polymyxin A1. Therefore, the addition of LPS could also play a role in inactivating the antimicrobial activity of polymyxins. Another explanation was about the two-component systems PhoP/PhoQ and PmrA/PmrB, which were involved in the LPS modifications and played an essential role in regulating polymyxins resistance. However, PhoP/PhoQ and PmrA/PmrB were reported to be affected by cation concentrations (calcium, iron, and magnesium) (Poirel et al., 2017).

Based on proteinaceous nature of antimicrobial agents, ammonium sulfate-graded precipitation, ion-exchange chromatography, and RP-HPLC were used for purification. The antimicrobial agents were mainly precipitated by 65∼100% saturation, which was speculated that the antimicrobial agents were more hydrophilic, requiring a higher saturation of ammonium sulfate (Guo et al., 2020). Subsequently, the antimicrobial substance was eluted from the CM Sepharose Fast Flow column with a high salt concentration of 60% buffer B. It was mainly attributed to the six cationic Dab residues making the polymyxin A1 positively charged at pH 7.4 (Velkov et al., 2016; Galea et al., 2017). The active peak C4, with a very weak UV absorption at 214 nm and no absorption at 280 nm, made it easy to be ignored in the separation process. This was mainly due to the absence of phenylalanine, tryptophan, and tyrosine in the structure of polymyxin A1, which made it difficult for purification. Another obstacle was the low molecular weight of polymyxin A1 (about 1156.7 Da), which invalidated the traditional concentration method by ultrafiltration tube during separation.

Polymyxin A1 was characterized by the mass and the structure by LC-MS, MALDI-TOF MS combined with whole genome mining. In this study, the mass of the active agent could be determined by LC-MS and MALDI-TOF MS. But it was always insufficient to elucidate the structure. The whole genome information provided us with a new reference for quickly focusing on the gene clusters involved in antimicrobial compounds (Zidour et al., 2019). This was due to the rapid development of diverse bioinformatics tools to mine genomic sequences for the biosynthetic gene clusters of ribosomal and non-ribosomal natural products (van Heel et al., 2017). A novel head-to-tail cyclized antimicrobial peptide was isolated and characterized from *Bacillus pumilus* by genome-guided identification (van Heel et al., 2017). Forty biosynthetic gene clusters were characterized in two cave strains of *Paenibacillus* sp. by genome mining (Lebedeva et al., 2020). Therefore, through biosynthetic gene clusters analysis, the molecular weight predicted by mass spectrometry combined with physicochemical assessment of the supernatant, we could deduce roughly what the active agent was. In addition, we also confirmed the inference of polymyxin A1 by LC-MS/MS, amino acid analysis, and antimicrobial assays in subsequent experiments.

From the antimicrobial spectrum of polymyxin A1 isolated, we found it mainly inhibited the Gram-negative bacteria, which was consistent with that of polymyxin antibiotics. Furthermore, an interesting observation was that the supernatant could also inhibit some Gram-positive bacteria but the polymyxin A1 isolated was invalid. This suggested that except for polymyxin A1, there may be other active agents produced in the supernatant of *P. thiaminolyticus* SY20. Many multiple-component bacteriocin systems are now known to be produced by Gram-positive organisms and have been reviewed recently (Martin et al., 2003). In the future, we can further explore whether *P. thiaminolyticus* SY20 produces other novel antimicrobial compounds. In addition, we found the supernatant and active agent had no inhibitory effect on its producing bacteria *P. thiaminolyticus* SY20, which may be attributed to the ORFs of *ctgl-5053* and *ctgl-5054* encoding membrane transporters. The locations of the two tandem transporters within the polymyxin A1 gene cluster suggest a role in conferring resistance against polymyxin *via* secretion by the producing cell (Choi et al., 2009; Galea et al., 2017).

The excellent antimicrobial activity of polymyxins against MDR pathogenic bacterial infections has led to its reemergence among the antibiotics currently used in clinical practice in order to deal with such bacteria. As the last resort to MDR, only polymyxin B and colistin (polymyxin E) are widely explored in polymyxins family. Although they share a common antimicrobial mechanism and spectrum, the only amino acid in their structure makes the activity and toxicity different (Kwa et al., 2007). Compared with polymyxin B and colistin, polymyxin A1 is structurally distinct by three and two amino acids, respectively. In further literature survey, we found polymyxin A1 was reported only in *Paenibacillus dendritiformis* and *Paenibacillus polymyxa*, which was a rarely explored member of polymyxins (Jangra et al., 2018).

## Conclusion

In this study, an antimicrobial strain, named *P. thiaminolyticus* SY20, was isolated from the sour bamboo shoots sample which possessed antimicrobial activity against both Gram-positive and Gram-negative foodborne pathogenic bacteria. The polymyxin A1 was purified and identified from the supernatant of *P. thiaminolyticus* SY20 that has never been reported in this species and is considered to have antimicrobial efficacy against Gram-negative bacteria. Meanwhile, the biosynthetic gene cluster of polymyxin A1 was determined, and the function of each ORF has been preliminarily identified and analyzed. Based on these findings, the polymyxin A1 produced by *P. thiaminolyticus* SY20 should be further explored for its applications.

## Data availability statement

The whole genome sequencing data of *P. thiaminolyticus* SY20 presented in the study was deposited in the NCBI repository with the accession number CP106992.

## Author contributions

Y-pW: investigation, formal analysis, and writing—original draft. Y-yH: writing—review and editing. JL and L-fX: editing. D-mL: writing—review, funding acquisition, and supervision. M-hL: writing—review. All authors have approved the final version to be published and agreed to be accountable for all aspects of the work in ensuring that questions related to the accuracy or integrity of any part of the work are appropriately investigated and resolved.

## Footnotes

1
https://antismash.secondarymetabolites.org

2
http://ab.inf.unituebingen.de/software/NRPSpredictor/

3
http://www.nrpssp.com/execute.php
