# Supplementary material for: Genome-guided purification and characterization of polymyxin A1 from Paenibacillus thiaminolyticus SY20: A rarely explored member of polymyxins
Source: Front Microbiol. 2022 Nov 14;13:962507. doi: 10.3389/fmicb.2022.962507 (PMC9701815; doi:10.3389/fmicb.2022.962507)
Supplement: Supplementary file 1 [file Data_Sheet_1.pdf]

Genome-guided purification and characterization of polymyxin A1 from *Paenibacillus thiaminolyticus* SY20: A rarely explored member of polymyxins

Ya-ping Wu[1,2], Dong-mei Liu[1*], Ming-hua Liang[1], Yan-yan Huang[1], Jin Lin[1], Lan-fang Xiao[1]

[1]School of Food Science and Engineering, South China University of Technology, Guangzhou 510640, Guangdong, China

[2]College of Food Engineering, Zhangzhou Institute of Technology, Zhangzhou 363000, Fujian, China

* Corresponding author:

Dong-mei Liu (E-mail: liudm@scut.edu.cn; Telphone:8620-222368198; Cellphone: 86-13609795325; Fax:8620-87113848)

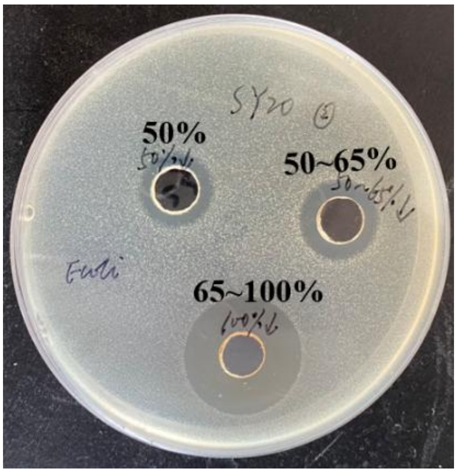

Fig.S1 The antimicrobial activity of the CE prepared by ammonium sulphate graded precipitation.The precipitations (precipitated by 65~100% saturation) exhibited the best antagonistic activity against *E. coli* ATCC 25922

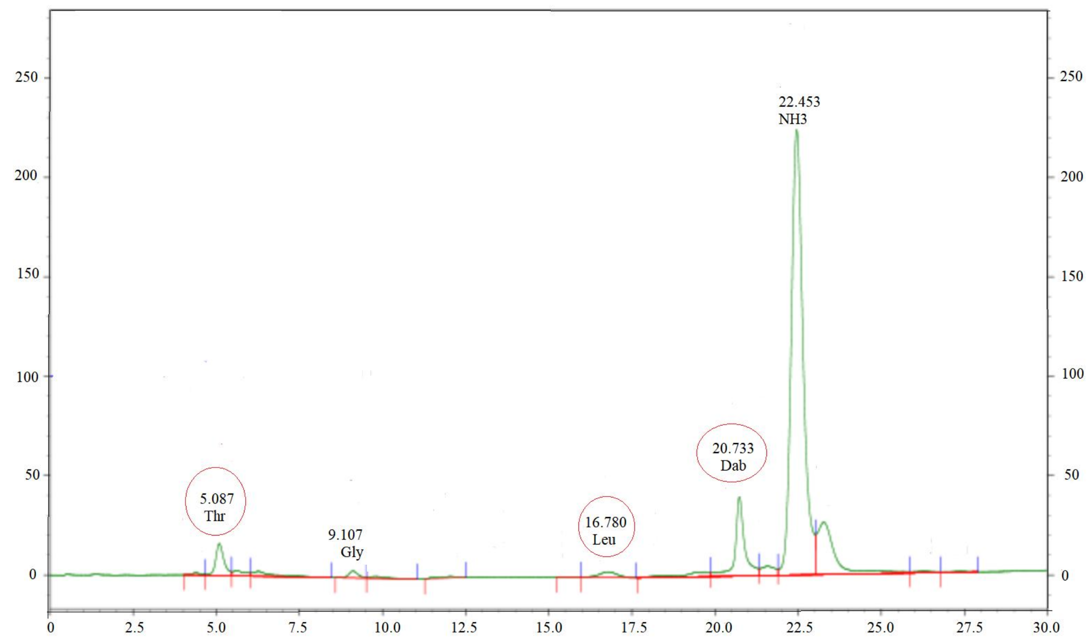

Fig.S2 The Amino acid composition of the active agent.Three amino acids Thr, Leu and Dab were detected, which is consistent with polymyxin A1

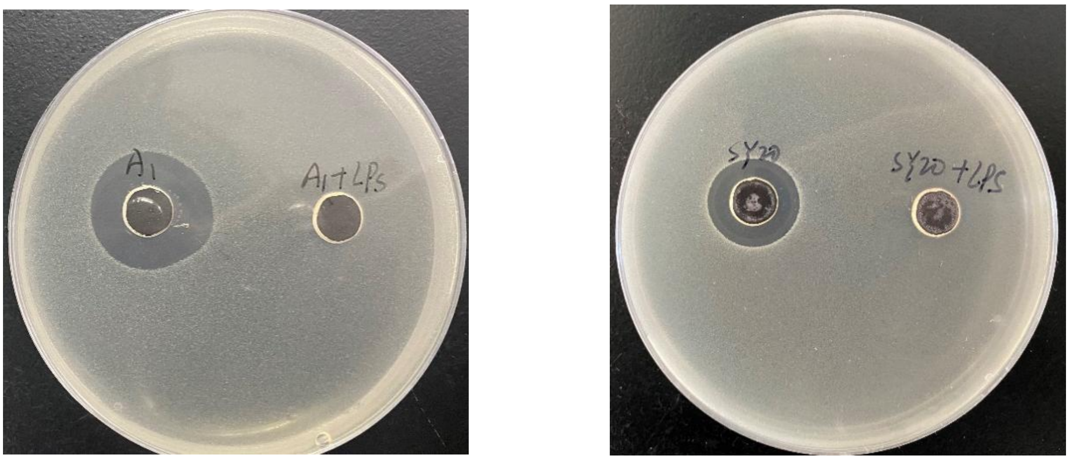

(A) **(B)**

Fig.S3 The antimicrobial activity of the pure antimicrobial agent with LPS (A) and the cell-free supernatant with LPS (B). The antimicrobial activity against *E. coli* ATCC 25922 disappeared when a certain amount of LPS were added to the active agent and the cell-free supernatant, respectively
