# Supplementary material for: Genome-guided purification and characterization of polymyxin A1 from Paenibacillus thiaminolyticus SY20: A rarely explored member of polymyxins
Source: Front Microbiol. 2022 Nov 14;13:962507. doi: 10.3389/fmicb.2022.962507 (PMC9701815; doi:10.3389/fmicb.2022.962507)
Supplement: Supplementary file 2 [file Table_1.pdf]

Genome-guided purification and characterization of polymyxin A1 from *Paenibacillus thiaminolyticus* SY20: A rarely explored member of polymyxins

Ya-ping Wu[1,2], Dong-mei Liu[1*], Ming-hua Liang[1], Yan-yan Huang[1], Jin Lin[1], Lan-fang Xiao[1]

[1]School of Food Science and Engineering, South China University of Technology, Guangzhou 510640, Guangdong, China

[2]College of Food Engineering, Zhangzhou Institute of Technology, Zhangzhou 363000, Fujian, China

* Corresponding author:

Dong-mei Liu (E-mail: liudm@scut.edu.cn; Telphone:8620-222368198; Cellphone: 86-13609795325; Fax:8620-87113848)

Table S1 The antimicrobial spectrum of the cell-free supernatant and the active agent

**Indicator bacteria**	**Mediums**	**Antimicrobial diameter (mm)**	
		**Supernatant**	**Active agent**
**Gram-negative bacteria**			
E. coli（ATCC 25922）	LB	19.4	23.0
Samomella enteritidis（CCTCC AB 94018）	LB	17.0	21.0
Klebsiella pneumoniae（ATCC 10031）	LB	18.3	20.4
Enterobacter sakazakii（ATCC 29544）	LB	16.0	20.1
Vibrio parahaemolyticu（ATCC10031）	BHI	17.2	18.2
Psychrobacter pulmonis	LB	19.1	23.8
Salmonella typhimurium (ATCC 14028)	LB	17.0	19.2
Pseudomonas aeruginosa PAO1	LB	18.2	19.8
Shewanella putrefaciens (ATCC 8071)	NB	—	—
Pseudomonas fluorescens (ATCC 13525)	NB	—	—
**Gram-positive bacteria**			
Bacillus subtilis	LB	16.1	—
Lactobacillus plantarum 9010	MRS	16.6	—
P.thiaminolyticus SY20	LB	—	—
Listeria monocytogene (ATCC 19115)	BHI		—
S. aureus RN4220	LB	18.2	
Lactobacillus rhamnosus B1107	MRS	17.8	—
Bacillus coagulans 13002	LB	—	—
Bacillus amyloliquefaciens K1	LB	—	—
Bacillus licheniformis SG18	LB	16.2	—

- represents that no antimicrobial activity is observed
